# How Important Is Vitamin D Supplementation in the Prevention of Exacerbations in Patients With Chronic Obstructive Pulmonary Disease (COPD): An Evidence-Based Review

**DOI:** 10.7759/cureus.79787

**Published:** 2025-02-27

**Authors:** Ludovina Rocha, Beatriz Figueiredo, Sara Eduarda Martins

**Affiliations:** 1 General and Family Medicine, Unidade de Saúde Familiar (USF) São Neutel, Unidade Local de Saúde de Trás-os-Montes e Alto Douro (ULSTMAD), Chaves, PRT

**Keywords:** copd, exacerbation, prevention, supplementation, vitamin d

## Abstract

This review examines the efficacy of vitamin D supplementation in preventing exacerbations in patients with chronic obstructive pulmonary disease (COPD), a major global contributor to morbidity and mortality. Exacerbations, often caused by infections, significantly worsen outcomes and elevate healthcare costs. Given its immunomodulatory properties, vitamin D has been hypothesized to reduce exacerbations, particularly in populations with vitamin D deficiency.

A systematic search of multiple databases was performed in February 2024 and evidence was synthesized from four systematic reviews with meta-analyses and one recent randomized controlled trial. Findings indicate that vitamin D supplementation does not generally reduce exacerbation rates in COPD patients. However, in patients with baseline 25-hydroxyvitamin D (25(OH)D) serum levels below 10 ng/mL, one meta-analysis reported a significant (p=0.006) reduction in moderate-to-severe exacerbations following supplementation.

Variability in study designs, dosages, and follow-up durations limit the generalizability of these findings. Nevertheless, clinical guidelines recommend screening and supplementing vitamin D in COPD patients, particularly those with severe deficiency. Clinicians should consider the low cost and risk of vitamin D supplementation and its potential benefits in reducing exacerbations in such patients.

## Introduction and background

Chronic obstructive pulmonary disease (COPD) is a major cause of morbidity and mortality, ranking among the top three causes of death worldwide. Approximately 210 million people suffer from COPD globally. In Portugal, the prevalence is 14.2% among individuals aged 40 years and older, with rates increasing with age and higher in males [1].

COPD is a heterogeneous disease characterized by chronic respiratory symptoms (dyspnea, cough, and sputum production) due to changes in the airway (bronchitis, bronchiolitis) and/or alveoli (emphysema) [1]. These changes result in persistent and often progressive and irreversible airflow obstruction [1,2]. This obstruction is linked to ciliary dysfunction and mucus hypersecretion, driven by an abnormal inflammatory response in the lungs [3]. Inflammatory cell infiltration and elevated levels of circulating and pulmonary pro-inflammatory cytokines are key characteristics of this process [2]. COPD results from a complex interaction between environmental and individual factors; however, active or passive exposure to tobacco smoke is the main risk factor for its development [1].

COPD exacerbations are strongly associated with an increased risk of respiratory and all-cause mortality [4]. Exacerbations are acute episodes lasting up to 14 days characterized by worsening dyspnea and/or cough and sputum production, which may also involve tachypnea and/or tachycardia, often associated with increased inflammation, both locally and systemically, triggered by airway infections, air pollution or other airway injuries [4]. Respiratory infections significantly contribute to COPD exacerbations, playing a role in approximately 78% of severe episodes [5]. These infections exacerbate airway inflammation, increasing both local and systemic inflammatory responses [6].

Vitamin D plays a crucial role in immune regulation and inflammatory responses [3]. It exhibits immunomodulatory properties in infectious diseases [5,7], promoting the differentiation of adaptive immune cells into a tolerogenic phenotype while suppressing pro-inflammatory mediators [7]. Additionally, it enhances phagocytosis and antimicrobial activity of innate immune cells [7]. In contrast, vitamin D deficiency in COPD patients is associated with reduced respiratory mucosal resistance, impaired immune function, and an increased risk of respiratory infections [3,8].

Since recent studies point to the efficacy of vitamin D in improving lung function [3] and given its associated functions, vitamin D supplementation has been hypothesized to reduce the exacerbation rate in COPD patients. Vitamin D deficiency is common in COPD patients [8], supplementing with a daily dose of 1,000 to 2,000 International Units (IU) of vitamin D3 may be necessary to maintain an optimal level of 25-hydroxyvitamin D (25(OH)D) ≥ 30 ng/mL [1]. Therefore, considering the low costs and risks involved in vitamin D supplementation [9], it makes sense to evaluate its effectiveness in reducing COPD exacerbations.

## Review

Methods

A comprehensive bibliographic search was conducted in February 2024 using the Medical Subject Headings (MeSH) terms: “Vitamin D” and “Pulmonary Disease, Chronic Obstructive”. Meta-analyses (MA) and Systematic Reviews (SR) were searched in online databases from The Cochrane Library, Database of Abstracts of Reviews of Effects (DARE), BMJ Evidence-Based Medicine, and PubMed between 2011 and 2024, in Portuguese and English. The search was supplemented by an additional query in PubMed for randomized controlled trials (RCTs) published between 2022 and 2024, using the same MeSH terms.

The population included individuals with COPD who received vitamin D supplementation. The independent variable was vitamin D intake, and the primary outcome was the reduction of COPD exacerbations.

Studies that did not address vitamin D supplementation and those that evaluated vitamin D supplementation for purposes other than preventing exacerbations were excluded, along with duplicates. The Strength of Recommendation Taxonomy (SORT) scale from the American Academy of Family Physicians was used to assign levels of evidence and strengths of recommendation [10].

Results

**Figure 1:**
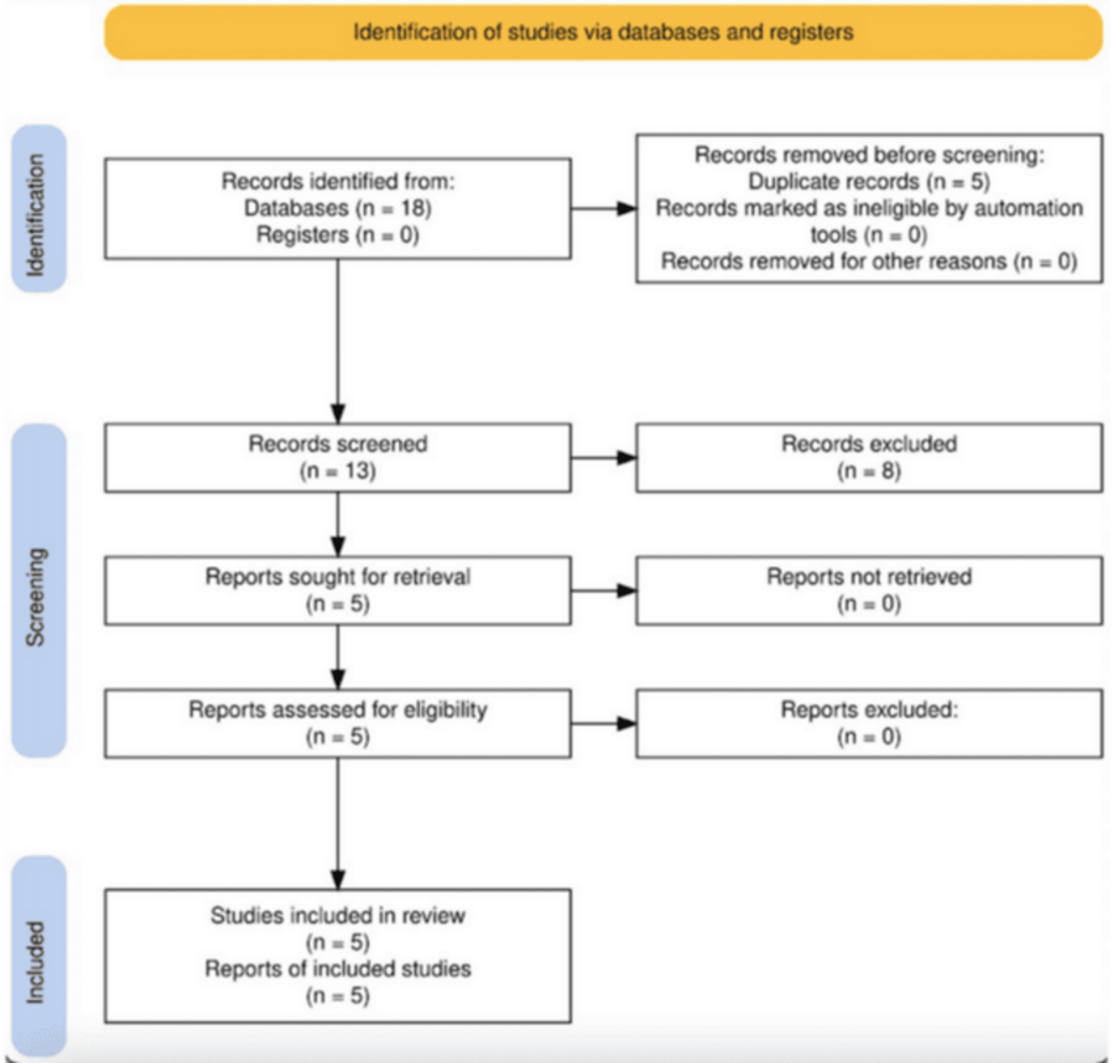
Articles selected Flow diagram prepared according to the PRISMA guidelines, available from https://www.prisma-statement.org/prisma-2020-flow-diagram.

From the 18 articles found in the databases, five duplicates were excluded. An additional eight were excluded, based on title and abstract, because they did not meet the inclusion criteria (Figure 1). Full-text reading of the remaining articles confirmed them for inclusion. The five articles included (four SR with MA and one RCT) are described in Table 1. In the articles selected for full reading, several results were obtained.

**Table 1:** Studies included in the present review COPD - Chronic obstructive pulmonary disease; RCT - Randomized Clinical Trial; MA - Meta-Analysis; N - Number *According to SORT definitions [10].

Reference	Type	Size	Vitamin D3 dosage	Duration of supplementation	Results	Level of evidence*
Hua et al. (2023) [3]	MA	5 RCT N=522	1,200 to 2,400 IU daily/50,000 to 100,000 IU single dose	6 days to 12 months	There is no apparent impact on the number of COPD exacerbations or any significant and robust lung function improvement.	2
Jolliffe et al. (2019) [6]	MA	3 RCT N=472	100,000 IU monthly/120,000 IU once every 2 months/1,200 IU daily	6 to 12 months	Vitamin D supplementation does not affect the overall rate of moderate-severe COPD exacerbations. Specific subgroup analysis revealed that vitamin D supplementation significantly reduced the rate of moderate-severe exacerbations in participants with baseline 25(OH)D levels <25nmol/L but not in participants with higher levels. Vitamin D levels should be tested in COPD patients who experience frequent exacerbations and supplementation should be offered to those with levels <25nmol/L.	2
Anitua et al. (2022) [5]	MA	5 RCT N=658	100,000 IU monthly/120,000 IU once every 2 months/50,000 IU weekly/1,200 to 2,000 IU daily	6 to 12 months	Vitamin D supplementation hasn’t shown a protective effect on the incidence of COPD exacerbations.	2
Rafiq et al. (2022) [7]	RCT	N=155	16,000 IU weekly	12 months	Vitamin D supplementation did not modify the 1-year exacerbation rate. There was no significant difference on the time until first and second exacerbations and time until hospitalizations. There was no difference in the use of antibiotics and corticosteroids, lung function, maximum respiratory pressure in the mouth, physical performance, skeletal muscle strength, inflammatory markers, composition of the nasal microbiota and quality of life.	2
Wang et al. (2022) [9]	MA	11 RCT N=1,183	600-50,000 IU daily/300,000 IU single injection	1 week to 12 months	Vitamin D supplementation appeared to decrease the number of patients with COPD exacerbations, however, this was not statistically significant. Vitamin D supplementation was a promising, low-cost, low-risk method for controlling COPD, especially in patients included in the subgroup vitamin D baseline < 25 nmol/L.	2

In a systematic review and meta-analysis by Jolliffe et al., that included three RCTs, vitamin D3 supplementation, and placebo were compared in 472 participants. In an RCT, vitamin D was administered in 12 monthly bolus doses for one year (total of 1,200,000 IU for one year); in another RCT, six bolus doses were administered every two months for one year (total of 720,000 IU for one year) and in another RCT one daily dose for six months (total of 220,000 IU for six months). Analysis of event rates allows for the inclusion of data from trials having different follow-up times. Prespecified subgroup analyses for the outcome of moderate or severe exacerbation rate were performed to identify factors modifying the effects of vitamin D supplementation [6].

The study found that vitamin D supplementation did not affect the overall frequency of moderate/severe COPD exacerbations. However, subgroup analysis indicated a protective effect in participants with baseline 25(OH)D levels below 25 nmol/L (p=0.006), whereas no benefit was observed in the remaining participants (p=0.71), with a p for interaction=0.015 [6]. Thus, it was concluded that vitamin D supplementation safely and substantially reduces the rate of moderate/severe COPD exacerbations in patients with baseline 25(OH)D levels <25 nmol/L, but not in those with higher levels [6]. Among participants randomized to the intervention arm of included studies, no difference in COPD exacerbation incidence was observed between participants who attained end-study serum 25(OH)D concentrations ≥75 nmol/L (n=138) versus those who did not (n=66) [6]. However, it is important to note that this systematic review with MA incorporates data from a small number of RCTs and the researchers were unable to obtain data from participants in an eligible RCT [6].

According to an MA by Hua et al., vitamin D supplementation, in patients with COPD, with 1,200-2,400 IU daily or 50,000-100,000 single doses (varying from six days up to 12 months of treatment), does not appear to reduce the number of exacerbations of the disease (p=0.724), nor did it show a significant and robust improvement in lung function [3]. The variation between vitamin D levels at baseline and end of treatment was not analyzed, as the authors noted that measurement methods were not standardized [3].

According to Anitua et al., in a systematic review and MA that included five randomized clinical trials on COPD exacerbations, vitamin D3 supplementation and placebo were compared in 658 participants. In one RCT, vitamin D was administered monthly, 2.5 mg bolus, for one year (total of 1,200,000 IU for one year); in another RCT, 3 mg bolus was administered every two months for one year (total of 720,000 IU for one year); in another RCT a weekly dose of 50,000 IU for six months and, in the other two RCTs, a daily dose of 1,200/2,000 IU for six months. The mean (and standard deviation) baseline serum 25(OH)D concentration was 49.8 (±29.2) nmol/L, 46.1 (±25.7) nmol/L; 47.2 (±12.2) nmol/L; 41.4 (±16.0) nmol/L; and 60.1 (±6.44) nmol/L, respectively. It was found that vitamin D supplementation did not demonstrate a protective effect on the incidence of COPD exacerbations (p=0.11) [6].

According to Rafiq et al., in a randomized clinical trial, a sample of 155 patients with COPD, aged 40 years or older, and at least one exacerbation in the previous year and vitamin D deficiency (25(OH)D 15-50 nmol/L), was randomly assigned to receive 16,800 IU of Vitamin D3 or placebo weekly for one year [7]. At baseline, the vitamin D group had a mean (and standard deviation) serum 25(OH)D concentration of 38 (±15) nmol/L, while the placebo group had 40 (±17) nmol/L [7]. After one year, serum 25(OH)D concentration was 112 (±34) nmol/L in the vitamin D group, compared with 42 (±17) nmol/L in the placebo group [7]. Vitamin D supplementation did not affect the exacerbation rate at one year (p=0.47) [7]. Also, no difference in exacerbation rate was observed in the subgroup of participants with 25(OH)D concentrations < 25 nmol/L [7]. There was no significant difference in the time interval between the first and second exacerbations and in the time to hospitalizations [7]. There was no difference in the use of antibiotics and corticosteroids, lung function, maximum respiratory pressure in the mouth, physical performance, skeletal muscle strength, inflammatory markers, nasal microbiota composition, and quality of life [7].

According to Wang et al., vitamin D supplementation improved COPD indicators, regarding lung function, in the group with severe vitamin D deficiency. They discuss results regarding 11 RCTs with heterogeneous vitamin D dose supplementation: seven trials with 2,000-3,500 IU daily (varying between eight weeks and 12 months of treatment), two trials with 600-1,200 IU daily (six to 12 months), one trial with 300,000 IU single injection (for one month) and one trial with 50,000 IU daily (for one week). Although the effect of treatment was heterogeneous among clinical trials and may have been overestimated, Vitamin D supplementation was a promising, low-cost, low-risk method for controlling COPD, especially in patients included in the subgroup vitamin D baseline < 25 nmol/L. In this subgroup of patients with vitamin D levels < 25 nmol/L, in six RCTs the end-of-treatment level of vitamin D was increased to 25-50 nmol/L [9]. Supplementation with vitamin D appeared to decrease the number of patients with COPD exacerbations (OR=0.90), however, this was not statistically significant (95%CI=(0.45, 1.77)). Their review evaluated RCTs with different methodologies, doses of vitamin D supplementation, and evaluation time during intervention (varying from one week to 12 months) and some RCTs lacked information regarding vitamin D baseline and end-of-treatment results in the patients studied [9].

Discussion

Interpretation of Results

Throughout the reviewed evidence, no overall reduction in the rate of COPD exacerbations has been observed as a result of Vitamin D supplementation in patients with the condition. However, there is one notable positive finding: in adults with both COPD and baseline serum 25(OH)D levels <10 ng/mL (<25 nmol/L), a reduction in exacerbations is associated with vitamin D supplementation.

These findings support or, at the very least, do not contradict the recommendations from many clinical guidelines (Table 2). Most guidelines converge in their recommendations regarding supplementation for vitamin D deficiency in COPD patients while acknowledging inconsistent evidence of benefit [11]. Notably, the aforementioned positive finding, by Jolliffe et al., has led some guidelines to recommend vitamin D supplementation in adults with COPD and severe vitamin D deficiency, defined as baseline serum 25(OH)D levels <10 ng/mL (<25 nmol/L) [2,4,11]. Additionally, a 2019 practice guideline recommends considering Vitamin D supplementation in adults with COPD with baseline serum levels of 25(OH)D 11 to 29 ng/mL (27.5 to 72.5 nmol/L), asserting that vitamin D supplementation in adults with COPD with serum 25(OH)D levels within this range may or may not improve lung function or reduce exacerbations [2]. However, the evidence we found did not provide support for such benefits in this range.

**Table 2:** Clinical guidelines COPD – chronic obstructive disease *According to SORT definitions [10].

Reference	Recommendation	Strength of recommendation*
Guia prático de gestão da DPOC nos cuidados de saúde primários (2021) [1]	It is recommended to screen for vitamin D deficiency in patients with COPD. Supplementing with a daily dose of 1000 to 2000 IU of vitamin D3 may be necessary to maintain an optimal level of 25 hydroxyvitamin D ≥ 30 ng/mL.	C
Chronic Obstructive Pulmonary Disease: A 2019 Evidence Analysis Center Evidence-Based Practice Guideline (2019) [2]	Assessment of the serum 25(OH)D level is recommended in adults with COPD with 2 or more exacerbations per year. Vitamin D supplementation is recommended in adults with COPD with baseline serum 25(OH)D levels <10 ng/mL (<25 nmol/L). Vitamin D supplementation in adults with COPD with baseline serum 25(OH)D levels <10 ng/mL (<25 nmol/L) has been shown to decrease exacerbations. Vitamin D supplementation should be considered in adults with COPD with baseline serum levels of 25(OH)D 11 to 29 ng/mL (27.5 to 72.5 nmol/L). Vitamin D supplementation in adults with COPD with serum 25(OH)D levels within this range may or may not improve lung function or reduce exacerbations.	B
COPD exacerbations: Prognosis, discharge planning, and prevention. UpToDate (2023) [4]	Vitamin D supplementation does not reduce the overall rate of moderate to severe COPD exacerbations, but a subgroup analysis revealed protective effects in patients with a baseline serum 25(OH)D level <10 ng/mL (<25 nmol/L).	B
Stable COPD: Overview of management. UpToDate (2023) [11]	Studies are underway to determine whether vitamin D supplementation in adults with COPD reduces the number of exacerbations and improves lung function, however at least one study has not identified a benefit.	C
Global Strategy for the diagnosis, management and prevention of Chronic Obstructive Pulmonary Disease (2024 Report) (2024) [12]	It is recommended to evaluate and investigate severe vitamin D deficits <10 ng/mL (<25nmol/L) in all patients hospitalized for COPD exacerbations, and should then be supplemented if necessary.	A
Malnutrition in advanced lung disease. UpToDate (2023) [13]	Vitamin D supplementation in patients with COPD did not demonstrate benefit in the studies evaluated.	A

Given the relevance of baseline levels to guide treatment, some guidelines recommend screening for vitamin D deficiency in patients with COPD. The GOLD 2024 Report recommends that all patients hospitalized for exacerbations should be assessed for severe vitamin D deficiency [12], while The Academy of Nutrition and Dietetics' 2019 guideline recommended assessment of the serum 25(OH)D level for adults with COPD with two or more exacerbations per year [2].

Limitations of Included Studies

The MA included in this review has limitations, such as the very small number of eligible RCTs and the lack of methodological homogeneity among them. Their data analysis often included studies with different methodologies, doses, and duration of vitamin D supplementation and follow-up duration. For example, while supplemented patients within each trial were exposed to the same dose, intervention groups across different trials were exposed to dissimilar vitamin D doses. This limits the measurement and generalizability of effect in aggregate data, as different regimens may have different degrees of effect. Furthermore, none of the studies specify how the therapeutic doses were chosen.

Another notable limitation is the short follow-up duration in many studies, with some observing patients for six months or less. In such cases, seasonal variations in vitamin D levels throughout the year were not accounted for. Moreover, some studies failed to report both baseline and post-treatment vitamin D levels, leaving key questions unanswered regarding the doses required to normalize serum concentrations in COPD patients and whether normalization correlates with improved clinical outcomes. Given that vitamin D levels tend to decrease in inflammatory states, it is likely that low levels do not solely indicate vitamin D deficiency but may also reflect the underlying inflammatory burden of COPD [13].

Limitations of the Review Methods

By including SR with MA, this article concludes the highest levels of the evidence hierarchy. However, this strength also imposes a limitation on their valuation, as different MA may use data from the same RCT, potentially skewing the conclusions. Furthermore, the search was limited to publications in Portuguese and English, which means newer evidence published in other languages may have been missed.

## Conclusions

The available evidence has implications for clinical practice, given the high prevalence of vitamin D deficiency among people with COPD. Those with serum 25(OH)D concentrations lower than 10 ng/mL may benefit from vitamin D supplementation. This supports a routine testing strategy for vitamin D status in COPD patients, especially those with a history of exacerbations, in order to identify those with severe deficiency and supplement them with an exacerbation-preemptive intent. In addition to the previously implied need for more RCTs, clinical guidelines would benefit from further research into factors that influence the effect of vitamin D supplementation in COPD, such as optimal dosage and patient factors that may elicit its protective effect on exacerbation rate.
